# Partial spleen embolization reduces the risk of portal hypertension-induced upper gastrointestinal bleeding in patients not eligible for TIPS implantation

**DOI:** 10.1371/journal.pone.0177401

**Published:** 2017-05-11

**Authors:** Matthias Buechter, Alisan Kahraman, Paul Manka, Guido Gerken, Alexander Dechêne, Ali Canbay, Axel Wetter, Lale Umutlu, Jens M. Theysohn

**Affiliations:** 1 Department of Gastroenterology and Hepatology, University Hospital Essen, Essen, Germany; 2 Institute of Hepatology London, Foundation for Liver Research, London, and Faculty of Life Sciences & Medicine, King’s College London, London, United Kingdom; 3 Department of Diagnostic and Interventional Radiology and Neuroradiology, University Hospital Essen, Essen, Germany; University of Sydney, AUSTRALIA

## Abstract

**Introduction:**

Upper gastrointestinal bleeding (UGIB) is a severe and life-threatening complication among patients with portal hypertension (PH). Covered transjugular intrahepatic portosystemic shunt (TIPS) is the treatment of choice for patients with refractory or recurrent UGIB despite pharmacological and endoscopic therapy. In some patients, TIPS implantation is not possible due to co-morbidity or vascular disorders. Spleen embolization (SE) may be a promising alternative in this setting.

**Materials and methods:**

We retrospectively analyzed 9 patients with PH-induced UGIB who underwent partial SE between 2012 and 2016. All patients met the following criteria: (i) upper gastrointestinal hemorrhage with primary or secondary failure of endoscopic interventions and (ii) TIPS implantation not possible. Each patient was followed for at least 6 months after embolization.

**Results:**

Five patients (56%) suffered from cirrhotic PH, 4 patients (44%) from non-cirrhotic PH. UGIB occured in terms of refractory hemorrhage from gastric varices (3/9; 33%), hemorrhage from esophageal varices (3/9; 33%), and finally, hemorrhage from portal-hypertensive gastropathy (3/9; 33%). None of the patients treated with partial SE experienced re-bleeding episodes or required blood transfusions during a total follow-up time of 159 months, including both patients with cirrhotic- and non-cirrhotic PH.

**Discussion:**

Partial SE, as a minimally invasive intervention with low procedure-associated complications, may be a valuable alternative for patients with recurrent PH-induced UGIB refractory to standard therapy.

## Introduction

Upper gastrointestinal bleeding (UGIB) is a severe and life-threatening complication among patients with portal hypertension (PH). Although the overall mortality has decreased over the last decades, recurrence rates can reach 70% and approximately 20%-50% of the patients do not survive UGIB [1,2]. The combination of pharmocological and endoscopic therapy is considered as first-line therapy in acute bleeding situations as well as for secondary prophylaxis. Covered transjugular intrahepatic portosystemic shunt (TIPS) is the treatment of choice for first-line treatment failure [3]. However, in some patients, TIPS implantation is (i) not possible due to contraindications (e.g., hepatic encephalopathy, advanced liver cirrhosis, congestive heart failure) or (ii) not feasible due to vascular disorders (e.g., splenic vein thrombosis) leading to sinistral PH without alteration of hepatic venous pressure gradient (HVPG) [4–6]. More invasive approaches to treatment of PH, such as shunt surgery (e.g., portocaval shunt, splenorenal shunt) or splenectomy, are effective as well [7–11]. However, surgical interventions are associated with relevant morbidity and mortality, as these patients frequently present with significant co-morbidities.

Spleen embolization (SE)—as a minimally invasive procedure—may be a promising alternative for prevention of UGIB among patients with PH when standard therapy fails. We therefore retrospectively analyzed 9 patients with refractory or recurrent PH-induced UGIB not eligible for TIPS implantation treated with SE at the University Hospital Essen between 2012 and 2016.

## Materials and methods

The University Hospital Essen ethics committee approved the retrospective, anonymous analysis of this data. The study has been conducted according to the principles expressed in the Declaration of Helsinki.

### Patients

Nine patients after PH-induced UGIB who underwent partial SE between 2012 and 2016 were included in the retrospective study. Cirrhotic PH was the main cause for UGIB. All patients met the following criteria: (i) gastrointestinal hemorrhage with primary or secondary failure of endoscopic interventions and (ii) TIPS implantation not possible.

### Spleen size and laboratory parameters

Spleen size was measured with a convex ultrasound probe. Laboratory parameters (serum bilirubin, INR, platelets, white blood cells, and hemoglobin) were determined one day before and six months after partial SE.

### Upper gastrointestinal endoscopy (UGE)

Endoscopically confirmed PH-induced UGIB episodes occuring before partial SE were analyzed. According to Baveno VI recommendations, upper gastrointestinal bleeding from esophageal varices was treated by band ligation, bleeding from gastric varices by cyanoacrylate injection, and bleeding from portal hypertensive gastropathy (PHG) by argon plasma coagulation (APC) [3]. UGE was performed by an endoscopist with an experience of > 1‘000 esophagogastroduodenoscopies.

### Spleen embolization

**Fig 1:**
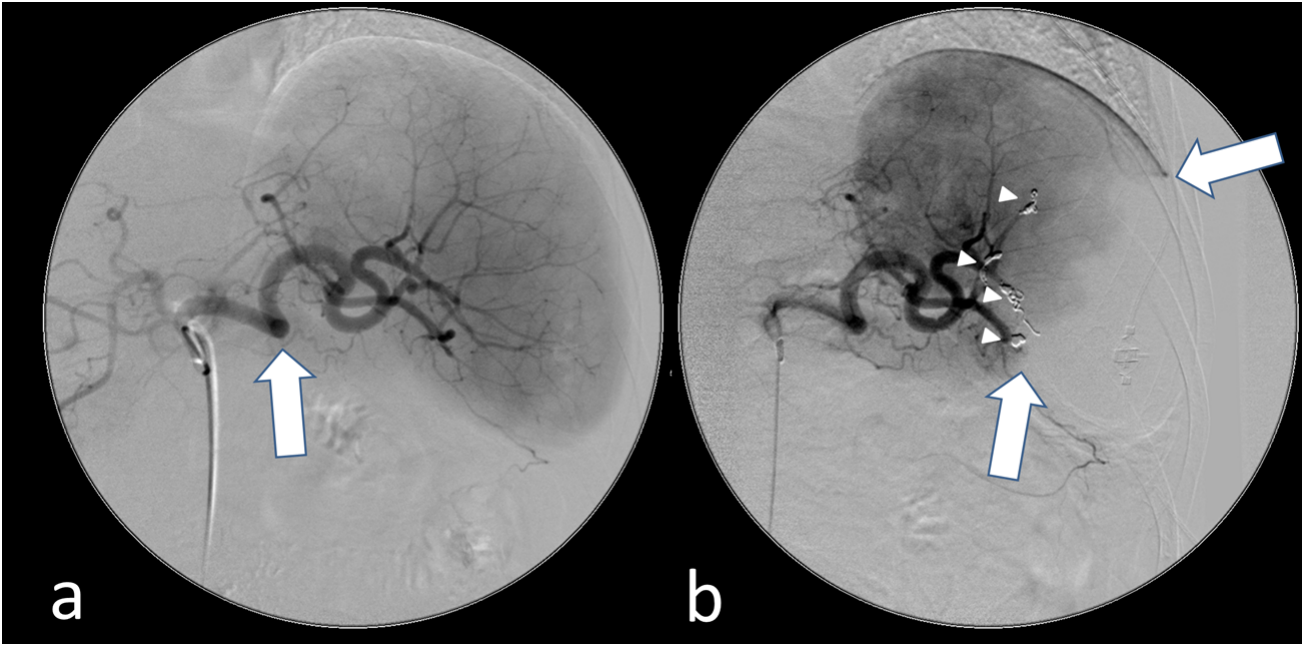
Partial coilembolization of the spleen. Angiographic images of the spleen with 5F-Sidewinder catheter in celiac trunk showing (a) the tortuous splenic artery (arrow) and the branching vessels before embolization and (b) the perfusion defect of approximately 60% (arrows) after coil embolization (arrow heads) of multiple peripheral arteries to the caudal pole of the spleen.

**Fig 2:**
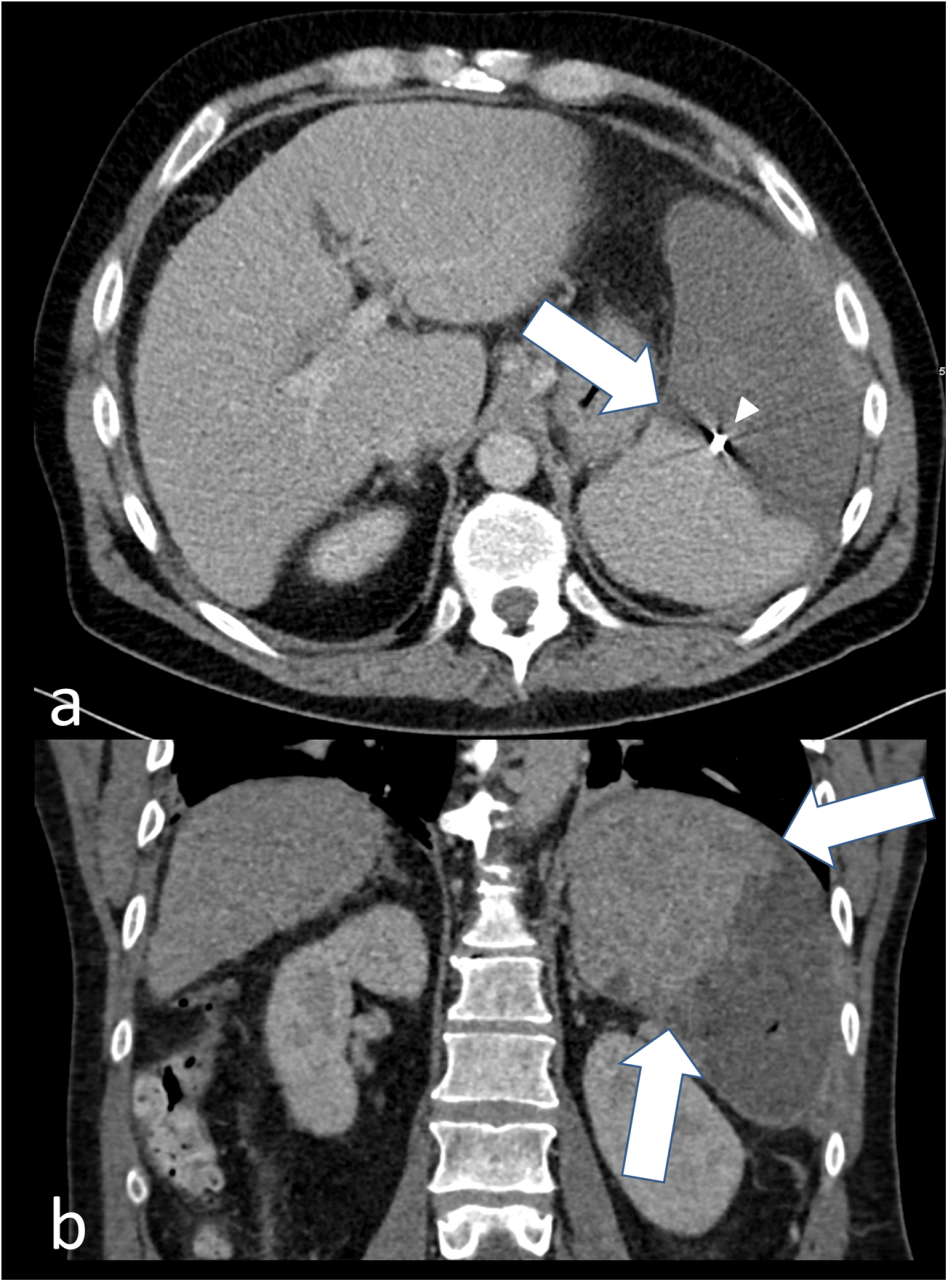
Partially devascularized spleen after coilembolization. Postinterventional CT images of the spleen in (a) transverse orientation with demarcation of parenchymal infarction (arrow) at the level of coil embolization (arrow head) and (b) in coronal orientation showing the demarcation zone (arrows) in correlation with the angiographic image (Fib 1b).

Splenic arteriography was performed on a biplane digital subtraction angiography (DSA) system (Philips Allura™, Philips Healthcare, Best, Netherlands or Toshiba Infinix DP-i, Toshiba Medical Systems, Tokio, Japan) via femoral artery access. Using a 5-French guiding catheter (Cobra 2, Sidewinder 1, or Sidewinder 2, Terumo Europe, Leuven, Belgium) contrast agent (Ultravist 300, Bayer Vital, Leverkusen, Germany) was administered in a dose of 15cc and an injection rate of 5cc / s utilizing an automatic injector (Medrad, Bayer Vital, Leverkusen, Germany). A microcatheter (Renegade, Boston Scientific, Natick, MA, USA; Rebar 18 or 27, Medtronic Inc., Minneapolis, MN, USA) was used to advance into the spleen vessels. Aiming to embolize 60% of the caudal pole of the spleen, peripheral branches were selectively cannulated and permanently occluded by coilembolization (IDC coils, Boston Scientific, Natick, MA, USA) or using Histoacryl (Braun Melsungen, Germany). Finally, angiography using the 5-French catheter was performed to document the result (**Fig 1**). Peri-interventional antibiotic prophylaxis was given with broad-spectrum antibiotics prior to the procedure and for two weeks post-procedural. Post-interventional CT scan was performed to document the immediate results (**Fig 2**).

### Follow-up examinations

Each patient was followed at least six months after partial SE for events of clinically significant UGIB which was defined by typical symptoms (melaena or hematemesis) with necessity of endoscopic treatment via UGE or red blood cell transfusions. In most patients UGE for surveillance of high-risk esophageal or gastric varices was routinely performed during six months after partial SE.

### Statistical analysis

Data are expressed as mean ± standard error of the mean (SEM). All variables were tested for normal distribution using the D'Agostino & Pearson omnibus and the Shapiro-Wilk normality tests. P values were calculated using unpaired Student t-test. A p value < 0.05 was considered statistically significant. Analysis was performed with Prism 6.0d (GraphPad Software, Inc, La Jolla, CA).

## Results

### Patient characteristics

Nine patients who underwent partial spleen embolization after recurrent UGIB from portal hypertension between 2012 and 2016 were included in the cohort. The mean age of the study population was 52.6 ± 19.1 [14–76] years. Five patients were male (56%). The mean Child-Pugh Score at the time of spleen embolization was 5.9 ± 1.3 [5–8] points, the mean MELD-Score 8.9 ± 2.6 [6–14]. All patients had splenomegaly with a mean spleen diameter of 16.6 ± 2.8 [13.3–22.3] cm. Each patient was followed for at least 6 months after embolization (mean survey time 17.7 ± 19.1 [6–52] months) (**Table 1**).

**Table 1:** Patient characteristics (n = 9).

Age, years		52.6 ± 19.1 [14–76]
**Gender**		
	**Male**	**5 (56%)**
	**Female**	**4 (44%)**
Etiology of PH*		
	AILD*	**3 (33%)**
	NAFLD*	**1 (11%)**
	HBV*-/HDV* **co-infection**	**1 (11%)**
	SV* **thrombosis**	**3 (33%)**
	PV* **thrombosis**	**1 (11%)**
**Bleeding location**		
	**Esophageal varices**	**3 (33%)**
	**Gastric varices**	**3 (33%)**
	PHG*	**3 (33%)**
**Child Pugh Score**		**5.9 ± 1.3 [5–8]**
**MELD Score**		**8.9 ± 2.6 [6–14]**
**Spleen size, cm**		**16.6 ± 2.8 [13.3–22.3]**
**Survey time, months**		**17.7 ± 19.1 [6–52]**

* AILD, alcohol-induced liver disease; NAFLD, non-alcoholic fatty liver disease; HBV, hepatitis B-virus; HDV, hepatitis D-virus; SV, splenic vein; PV, portal vein; PHG, portal-hypertensive gastropathy; PH, portal hypertension.

### Etiology of PH-induced UGIB and endoscopic findings

Liver cirrhosis with PH was the main cause for UGIB (alcohol-induced cirrhosis (3/9; 33%), non-alcoholic fatty liver disease (1/9; 11%), chronic hepatitis B- and D virus infection (1/9; 11%)) while four patients suffered from non-cirrhotic PH (idiopathic thombosis of the splenic- (2/9; 22%) or portal vein (1/9; 11%), thrombosis of the splenic- and superior mesenteric vein caused by chronic pancreatitis (1/9; 11%)). Endoscopic classification of PH-induced UGIB were in detail: hemorrhage from gastric varices (3/9; 33%), hemorrhage from esophageal varices (3/9; 33%), and hemorrhage from PHG (3/9; 33%) (**Table 1**).

### Contraindications to TIPS placement

None of the patients was suitable for TIPS placement because of the following criteria: congestive heart failure in 4 (44%), sinistral PH due to splenic vein thrombosis in 3 (33%), thrombosis of the portal vein with cavernous transformation in one (11%), and hepatic encephalopathy in one patient (11%).

### Additional endoscopic and pharmacological therapy among patients who underwent partial SE

All patients received non-selective beta-blockers (NSBB; propranolol or carvedilol) for secondary prophylaxis of UGIB. Two patients (22%) were additionally treated with repeated EVL after SE, 7 patients (78%) had no additional endoscopic prophylaxis.

### Partial SE led to increase of blood cell counts while liver function remained stable

Laboratory parameters (platelets, white blood cells, hemoglobin, bilirubin, and INR) were measured before and 6 months after SE. Partial SE led to increase in the numbers of platelets (pre: 70.89 ± 34.87 [34–132] x 10^3^/μl, post: 121.44 ± 42.49 [77–196] x 10^3^/μl, p < 0.05), white blood cells (pre: 3.17 ± 1.94 [1.23–7.18] x 10^3^/μl, post: 4.83 ± 2.02 [2.64–9.10] x 10^3^/μl, p = 0.09), and hemoglobin (pre: 10.09 ± 1.50 [8.10–12.60] g/dl, post: 10.87 ± 1.88 [8.20–13.70] g/dl, p = n.s.); only changes in platelet count were statistically significant while white blood cell counts showed an increasing trend. Liver function, displayed by total serum bilirubin (pre: 0.66 ± 0.27 [0.30–1.00] g/dl, post: 0.54 ± 0.19 [0.20–0.80] g/dl, p = n.s.) and INR (pre: 1.09 ± 0.07 [0.99–1.21], post: 1.06 ± 0.09 [0.92–1.24], p = n.s.) remained unchanged after partial SE. Laboratory parameters are demonstrated in **Table 2**.

**Table 2:** Laboratory data before and six months after partial spleen embolization.

	Pre SE*	Post SE*	p
**Platelets, x 10**^**3**^**/μl**	**70.89 ± 34.87 [34–132]**	**121.44 ± 42.49 [77–196]**	**< 0.05**
**White blood cells, x 10**^**3**^**/μl**	**3.17 ± 1.94 [1.23–7.18]**	**4.83 ± 2.02 [2.64–9.10]**	**0.09**
**Hemoglobin, g/dl**	**10.09 ± 1.50 [8.10–12.60]**	**10.87 ± 1.88 [8.20–13.70]**	**n.s.**
**Total bilirubin, mg/dl**	**0.66 ± 0.27 [0.30–1.00]**	**0.54 ± 0.19 [0.20–0.80]**	**n.s.**
**INR**	**1.09 ± 0.07 [0.99–1.21]**	**1.06 ± 0.09 [0.92–1.24]**	**n.s.**

* SE, spleen embolization

### Partial SE was successful in all cases and was associated with few procedure-associated complications

Partial SE was technically successful in all patients. Post embolization syndrome (PES), defined as a combination of fever, nausea, and abdominal pain without evidence for infection, occured in 6/9 patients (67%) and was controllable with analgetics (e.g., metamizole, opiates) and/or antiemetics (e.g., metoclopramide, dimenhydrinate) in all cases. Prolonged fever, defined as lasting more than 7 days after the procedure, did not occur. The mean postinterventional hospital stay after partial SE was 6 ± 2 [2–8] days. None of the patients suffered from major complications attributed to SE (e.g., splenic abscess, sepsis, splenic rupture, or pneumonia).

### Partial SE alone or in combination with EVL reduced the risk of portal hypertension-induced upper gastrointestinal re-bleeding

During cumulative follow-up of 159 (17.7 ± 19.1 [6–52]) months, none of the patients died, none required additional blood transfusions or experienced clinically relevant upper gastrointestinal re-bleeding (defined by endoscopic treatment for acute hemorrhage or blood transfusions).

**Fig 3:**
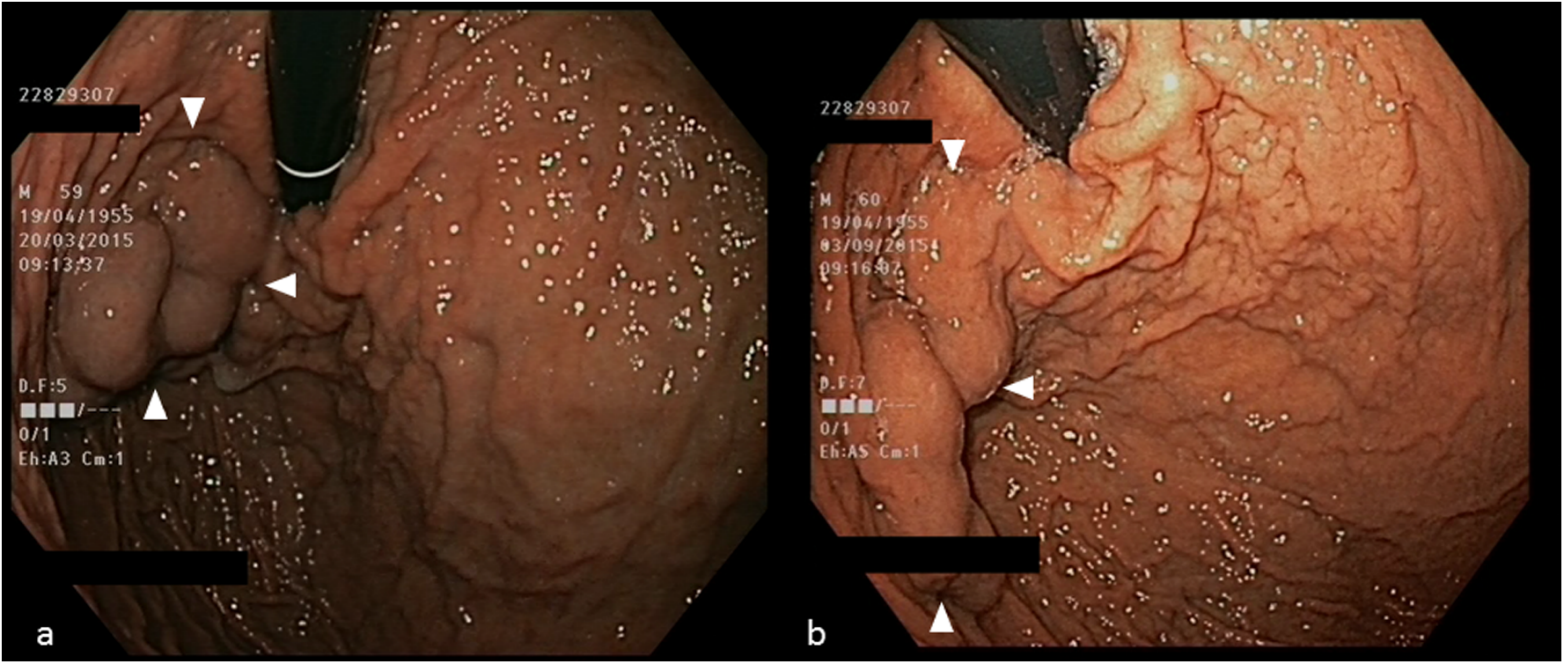
**Esophagogastroduodenoscopy showing (a) large-sized gastric varices in the gastric fundus before partial splenic embolization (SE) and (b) distinct regression of varices (arrows) six months after partial SE**.

**Fig 4:**
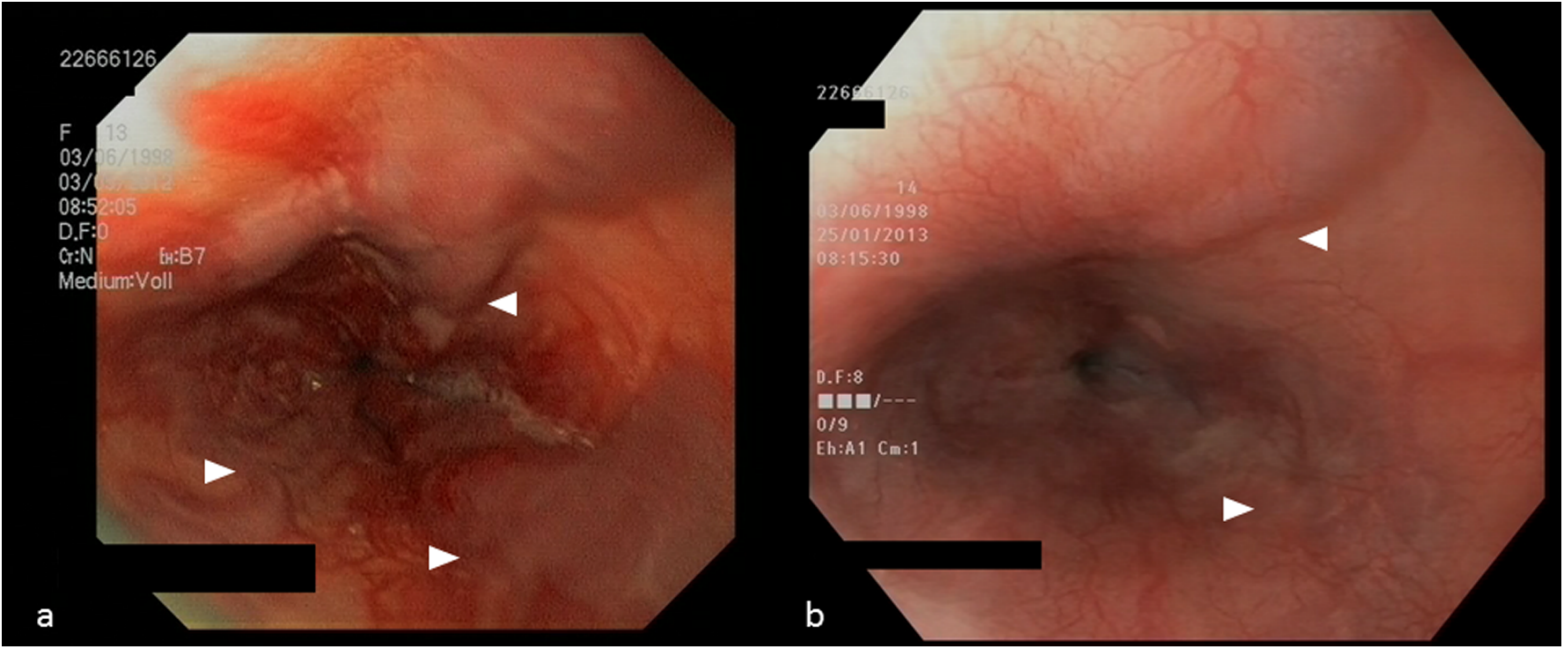
**Esophagogastroduodenoscopy showing (a) large-sized esophageal varices before partial splenic embolization (SE) and (b) distinct regression of varices (arrows) with scarring of the squamous epithelium eight months after partial SE in combination with endoscopic variceal ligation**.

Follow-up UGE examinations were performed in 6/9 patients (66.7%). None of the patients demonstrated signs of (active or preceding) bleeding and there was no evidence of progression of PH (**Figs 3 and 4**). 3/9 patients (33.3%) did not undergo follow-up UGE.

## Discussion

Patients with chronic liver disease and clinically significant PH, defined by HVPG ≥ 10 mmHg, are at risk of developing gastroesophageal varices. Despite improvements in diagnostics and therapy over the last decades, variceal bleeding still is life-threatening with a 6-week mortality of 10–20% [2,12,13]. In this regard, recent studies indicate that transient elastography is a promising non-invasive screening method for detecting decompensated PH, suggesting a direct positive correlation between spleen and liver stiffness and esophageal variceal bleeding [14,15].

PH-induced UGIB can occur as hemorrhage from PHG, esophageal- or gastric varices. Apart from that, vascular disease can lead to PH in case of thrombosis of the portal-, mesenteric-, or splenic vein (non-cirrhotic PH) [16,17]. Splenic vein thrombosis leads to sinistral (segmental or left-sided) PH confined to the left-sided part of the portal venous system [18]. Unlike cirrhotic PH, sinistral PH is characterized by a patent portal vein, normal hepatic function and unaltered HVPG [6,19,20].

According to Baveno VI consensus, management of acute bleeding includes pharmacological (antibiotic prophylaxis, prevention of hepatic encephalopathy, and application of vasoactive drugs (e.g., terlipressin, somatostatin)) and endoscopic therapy (endoscopic variceal ligation (EVL) for esophageal varices, therapy with tissue adhesive (e.g., cyanoacrylate) for gastric varices). For secondary prophylaxis, the combination of NSBB (propranolol or carvedilol) and EVL is recommended [3].

TIPS is the gold standard for PH-induced UGIB refractory to endoscopic and pharmacological therapy or for patients who present at high risk of secondary treatment failure (e.g., Child Pugh class C < 14 points or Child Pugh class B with active bleeding) [3,21–24]. Management of patients with contraindications to TIPS implantation or sinistral PH and regular HVPG is still under debate as generally accepted recommendations do not exist.

Balloon-occluded retrograde transvenous obliteration (BRTO) has gained popularity over the last decades showing considerable effectiveness in controlling gastric variceal bleeding with low re-bleeding rates [25,26]. It is an endovascular technique requiring a large infradiaphragmatic left-sided portosystemic collateral (usually a gastrorenal shunt) which provides venous outflow into gastric varices and is usually accessed from a transjugular or transfemoral vein [27,28]. During BRTO procedure, the gastrorenal shunt is occluded by an occlusion balloon followed by the endovascular injection of a sclerosing agent (e.g., oldamin, sotradecol, polidocanol) directly into the gastro-variceal system [27,29]. BRTO is a minimal-invasive procedure which can be performed in patients with poor hepatic function with or without hepatic encephalopathy and is even considered to improve both [25,26,30]. However, BRTO obliterates portosystemic (TIPS equivalent) shunts, potentially aggravating portal hypertension and its related complications resulting in progression of esophageal varices and ascites [31–33]. In expert opinion, role of BRTO in the management of gastric variceal bleeding is promising but merits further evaluation [34].

Splenectomy is another therapeutic option in this setting [6,35]. However, it is an invasive procedure with several disadvantages, as patients with PH induced UGIB frequently present in conditions unfit for surgery. Partial SE is a technique used for the mitigation of PH to reduce the risk of UGIB [36,37]. Several studies indicate that partial embolization of the spleen with or without variceal ligation significantly reduces variceal rebleeding. Ohmoto et al. described 52 cirrhotic patients with UGIB and compared bleeding rates after SE in combination with EVL to EVL alone, showing a significant reduction of re-bleeding in follow-up from 39% to 12% [38]. Likewise, Taniai et al. displayed a reduction of re-bleeding rate from 58% to 21% between both groups in identical study design [39]. Xu and colleagues treated 41 patients with esophageal variceal bleeding by combination of EVL and partial SE; only one patient suffered from recurrent bleeding (2.4%) [40]. Pälsson et al. reported a decrease of bleeding episodes from 4.3% to 1.1% in patients with liver cirrhosis, esophageal varices and thrombocytopenia treated with partial SE [41]. In a review of 5 studies including patients with PH, Koconis and co-authors demonstrated a reduction in bleeding episodes from 2.4 to 0.48 per year after SE [42]. Finally, Shimizu et al. described the successful treatment of a critically ill patient with refractory bleeding from PHG not eligible for TIPS placement [43].

We report on a cohort of 9 patients with recurrent UGIB due to PH. The combination of endoscopic and pharmacological therapy failed to permanently control the bleeding in all cases. TIPS implantation was contraindicated (in patients with cirrhotic PH) or patients suffered from sinistral PH caused by splenic vein thrombosis (patients with non-cirrhotic PH) where TIPS is not a treatment option. In accordance with published data, SE was successful and safe in all treated patients. The most common complication was the post embolization syndrome (PES) occurring in 6/9 patients (67%). These findings match well with the results of Gu et al., who performed SE for hypersplenism in 49 cirrhotic patients, and reported a PES rate of 75% [44]. None of our patients experienced major complications. Liver function, determined by serum bilirubin and INR, remained stable after partial SE. However, statistically significant improvement of liver function tests, as described in literature, were not found among our patients on 6 month follow-up examinations [36,45]. Hematological changes following SE are well described in literature and SE is reported to be a reasonable therapeutic approach among patients with severe thrombocytopenia [46–48]. As expected, SE led to significant increase in platelet count in our patients on 6 month follow-up examination.

We sought to investigate whether partial SE reduces the risk of PH-induced UGIB among patients who (i) present with gastrointestinal hemorrhage with primary or secondary failure of endoscopic interventions and (ii) are not eligible for TIPS implantation. None of the patients, treated with partial SE and meeting above mentioned criteria, experienced re-bleeding episodes, required blood transfusions or demonstrated progression of PH on UGE during a total follow-up time of 159 months, including both patients with cirrhotic PH and non-cirrhotic PH. Undoubtedly, our study has certain limitations since cohort size was small and analysis was performed retrospectively via chart-review. Nevertheless, we believe that partial SE, as a minimal invasive intervention with few procedure-associated complications, may be a valuable alternative in patients with recurrent or refractory PH-induced UGIB not eligible for TIPS.
